# Prevalent and diverse new plasmid-encoded heavy metal and antimicrobial resistance in *Klebsiella* strains isolated from hospital wastewater

**DOI:** 10.3389/fcimb.2025.1653886

**Published:** 2025-11-25

**Authors:** Grace Pascale Engobo, Wenya Su, Shengyao Wang, Zhen Yan, Youming Zhang, Mengge Zhang, Xueyun Geng, Hai Xu, Ling Li, Mingyu Wang

**Affiliations:** 1 State Key Laboratory of Microbial Technology, Microbial Technology Institute, Shandong University, Qingdao, China; 2 School of Environmental Science and Engineering, Shandong University, Qingdao, China; 3 Qilu Hospital Qingdao, Cheeloo College of Medicine, Shandong University, Qingdao, China; 4 Shanghai Key Laboratory of Atmospheric Particle Pollution and Prevention (LAP), Shanghai, China

**Keywords:** *Klebsiella*, plasmid, hospital wastewater, metal resistance, antimicrobial resistance, integron

## Abstract

The rise of antibiotic-resistant *Klebsiella pneumoniae* poses a significant global health threat. Plasmids, as mobile genetic elements, play a critical role in bacterial adaptation by facilitating the spread of resistance genes. To analyze plasmid-mediated antibiotic and heavy metal resistance in clinical *Klebsiella* strains, 33 *Klebsiella* strains isolated from wastewater were subjected to third-generation nanopore sequencing to obtain high-quality whole-genome assemblies. The presence and diversity of plasmids associated with antibiotic and heavy metal resistance were analyzed, and phenotypic assays were conducted to confirm metal resistance. A total of 81 plasmids were identified across 24 strains, including 28 (34.6%) novel plasmids. Among them, 22 plasmids carried antibiotic resistance genes (ARGs), with 12 containing integrons, four of which were complex Class I integrons and two unconventional integrons. Notably, a novel conjugative plasmid, pKP228-1, was discovered carrying a complex Class I integron with a unique gene cassette array encoding 12 ARGs, and harboring *bla*_NDM-1_ in the adjacent IS*CR1*-associated region. Another plasmid, pKP174-2, harbored *mcr-8.1* and *tporJ1-tmexCD1*. Additionally, 24 plasmids encoded resistance to eight heavy metals/metalloids, and 12 plasmids co-harbored both ARGs and metal resistance genes, indicating potential co-selection mechanisms. This study highlights the extensive diversity and novel structures of plasmids carrying both antibiotic and heavy metal resistance in clinical *Klebsiella* isolates. The observed co-occurrence of the two resistance types highlights the need for comprehensive genomic surveillance to monitor the spread of multi-resistance determinants.

## 1 Introduction

In recent years, the increasing prevalence of antibiotic-resistant bacteria has become a significant threat to public health worldwide. *Klebsiella* species, particularly *Klebsiella pneumoniae*, have emerged as critical opportunistic pathogens accountable for a wide range of infections associated with healthcare settings (Prestinaci et al., 2015; Tacconelli et al., 2018). *K. pneumoniae* is a prevalent Gram-negative bacteria belonging to the *Enterobacteriaceae* family are responsible for various illnesses, including pneumonia, bacteremia, liver abscess, and urinary tract infections (Navon-Venezia et al., 2017). This bacterium is also known for its high level of antibiotic resistance, which has significantly impacted the treatment of its infections.

The primary reason for multidrug resistance of *Klebsiella* strains is its accumulation of antibiotic-resistant plasmids (Li et al., 2018). Plasmids allow for the horizontal transfer of resistance traits between bacterial strains (Giraud et al., 2017; Rozwandowicz et al., 2017; Su et al., 2024). Certain antibiotic-resistant bacteria can harbor several plasmids, facilitating the exchange of antibiotic-resistance genes (ARGs) among them (Weingarten et al., 2018). It has been recognized that ARG transfer across plasmids is widespread, and 87% of ARGs were identified to transfer among distinct plasmids among 8,229 plasmid-borne ARGs (Wang et al., 2024a). In hospital settings, the transfer of antibiotic-resistant plasmids between bacteria is accelerated under the selective pressure exerted by antibiotics in the environment, which contributes to the emergence of highly antibiotic resistant pathogens (Li et al., 2022).

In addition to antibiotics, heavy metals are also known to contaminate hospital wastewater (Emmanuel et al., 2009). This is likely caused by the frequent pharmaceutical use of heavy metals. For instance, silver is often used as a disinfectant (Silvestry-Rodriguez et al., 2007), whereas mercury is commonly used as tooth fillings (Pradhan and Srivastava, 2022). Studies have shown that hospital effluents can contain both heavy metals and pharmaceuticals, as observed in wastewater from Indonesian hospitals during the COVID-19 pandemic (Sakina et al., 2023), highlighting potential environmental and health risks. Therefore, clinical *Klebsiella* strains are often found to be heavy metal-resistant or heavy metal-tolerant (Radisic et al., 2024). Whether the emergence of heavy metal resistance is also driven similarly as antibiotic resistance, aka by accumulation of resistance plasmids, still needs further investigations.

Surveillance of plasmids in *Klebsiella* strains has shown that a high percentage of detected plasmids are new (Wang et al., 2024b; Xu et al., 2024). This can be attributed to the higher level of variability of plasmids due to carriage of recombination-related genes, as well as the lack of affordable sequencing technologies that can reliably detect and sequence whole plasmids. Application of 3^rd^ generation sequencing technologies, with the fast decrease of sequencing costs, has enabled cost-effective detection of plasmids. Therefore, we are now at a position to better understand plasmid-mediated antibiotic and heavy metal resistance in *Klebsiella* and other pathogenic bacteria.

This work aims to analyze plasmid-encoded antibiotic and heavy metal resistance in *Klebsiella* strains in a clinical setting. Specifically, we investigated (1) the role of plasmids in heavy metal resistance, (2) the co-occurrence between antibiotic and heavy metal resistance, and (3) the occurrence and characteristics of newly identified heavy metal-resistant plasmids similar to antibiotic-resistant plasmids.

## 2 Materials and methods

### 2.1 Bacterial strains

*Klebsiella* spp. used in this work were isolated from wastewater collected from a wastewater treatment facility between 2019 and 2020 from Qilu Hospital in Qingdao, China, as described in our previous publication (Li et al., 2022). *Klebsiella* strains were purified by growth overnight at 37°C on MacConkey agar plates without antibiotics.

### 2.2 Antimicrobial susceptibility testing

Antibiotic MICs were determined using the broth microdilution method in 96-well plates, following the Clinical and Laboratory Standards Institute (CLSI) and the European Committee on Antimicrobial Susceptibility Testing (EUCAST) guidelines. The antibiotics tested included Imipenem, Tigecycline, Polymyxin E, Ampicillin, Cefotaxime, Kanamycin, Streptomycin, Trimethoprim, Tetracycline, Ciprofloxacin, Gatifloxacin, Meropenem, and Sulfisoxazole. *Escherichia coli* ATCC 25922 was used as a quality control strain for antimicrobial susceptibility testing.

### 2.3 DNA extraction and whole-genome sequencing

The genomic DNA of *Klebsiella* strains was extracted using the TIANamp DNA Kit (Tiangen Biotech (Beijing) Co., Ltd., Beijing, China) according to the manufacturer’s instructions. The purity and quantity of DNA were determined using a Qubit™ 4.0 fluorometer (Thermo Fisher Scientific, MA, US). The sequencing library was constructed from 150 ng of genomic DNA using the Oxford Nanopore rapid barcoding kit SQK-RBK114.96. It was sequenced on the Nanopore p2solo platform (Oxford Nanopore Technologies, Oxford, UK) with an R10.4.1 flow cell. To obtain raw data, Basecalling was performed using Dorado version 0.5.3 (https://github.com/nanoporetech/dorado/).

### 2.4 Bioinformatics

The analysis of whole-genome sequencing (WGS) data for all isolates was conducted utilizing various bioinformatics tools. Flye version 2.8.1-b1676 was employed to assemble the genomes from long reads (Kolmogorov et al., 2019) and to determine sequence circularity. Additionally, Quast version 5.0.2 and CheckM2 version 1.0.2 were utilized to assess the assembly’s quality and completeness and check for contamination (Mikheenko et al., 2018; Chklovski et al., 2023). GTDB-Tk version 2.1.1 was used to determine the taxonomic classification of genomes. The genomes were annotated with the Prokaryotic Genome Annotation Pipleline (Tatusova et al., 2016). AMRFinder version 3.11.26 and Plasmidfinder version 2.1.1 were utilized to identify antimicrobial resistance genes (ARGs) and plasmid replicon types (Carattoli et al., 2014; Feldgarden et al., 2019). Multilocus sequence typing (MLST) and serotypes were identified using kleborate.

### 2.5 Quality control and assembly assessment

Raw reads were subjected to quality control using fastp (v0.23.4). Adapter sequences and low-quality bases (Q < 20) were trimmed, and reads shorter than 500 bp after trimming were removed. The quality of clean reads was checked with FastQC, ensuring that >90% of bases reached Q30. Genome assemblies were subsequently evaluated with QUAST (v5.2.0) to obtain metrics including N50, GC content, and genome size. Assembly completeness and contamination were assessed using CheckM (v1.0.2).

### 2.6 Metal resistance assays

Heavy metal resistance assays were performed by agar dilution using Mueller-Hinton Agar plates. Overnight cultures of strains were diluted and adjusted to an OD_600_ value of 0.08–0.1 using LB medium. A 5 µL aliquot of the diluted cultures was inoculated onto MH Agar plates containing various concentrations of metals. Metal salts, including K_2_TeO_3_, CuSO_4_, CoCl_2_, AgNO_3_, K_2_Cr_2_O_7_, NiSO_4_, and HgCl_2_, were added to the media, and the plates were incubated at 37 °C for 24 hours. Experiments were performed with three (n=3) independent technical replicates. *K. pneumoniae* ATCC13883 was used as the control strain.

## 3 Results and discussion

### 3.1 *Klebsiella* strains isolated from hospital wastewater and whole genome sequencing

Thirty-three *Klebsiella* isolates were obtained from the wastewater of Qilu Hospital Qingdao as part of a large-scale surveillance of wastewater bacterial communities previously reported by our group (Li et al., 2022). Four of the isolates, 1-76, 2-28, 3-82, and 3-88, are *Klebsiella quasipneumoniae* strains, while the remaining strains are *K. pneumoniae* strains. Third generation Nanopore sequencing was performed to obtain the whole genome sequences of these isolates. The long reads of this technology led to the assembly of high quality genomes, with the generation of near-perfect plasmid maps. All 33 isolates have their chromosomes well assembled to their circular forms, with the sizes of 5.34 ± 0.11 Mb, in agreement with the common sizes of *Klebsiella* chromosomes. Only four of the isolates were free of plasmids, all of which are *K. pneumoniae*. Analysis of plasmid-containing *Klebsiella* isolates suggested that they belong to 24 genomically distinct strains, as determined by whole-genome sequence similarity and assigned LIN codes using the Pathogenwatch cgMLST-based classification system (**Supplementary Table 1**). These strains were subject to further studies. Features of these strains are documented in **Table 1**. These strains belong to various and diverse sequence types and serotypes, showing high levels of heterogeneity. On average, they carry 3.4 plasmids per strain.

**Table 1:** Studied strains in this work.

Strain	Species	Sequence type	K-serotype	O-serotype	Number of plasmids
1-74	*K. pneumoniae*	ST4508-1LV	KL185	O1/O2v1	5
1-76	*K. quasipneumoniae*	ST5435	KL72	O12	5
2-28	*K. quasipneumoniae*	ST1308	KL120	OL103	3
2-55	*K. pneumoniae*	ST37	KL23	O1/O2v2	2
2-59	*K. pneumoniae*	ST147	KL81	OL13	3
2-61	*K. pneumoniae*	ST29	KL54	O1/O2v2	4
2-65	*K. pneumoniae*	ST1	KL19	O1/O2v2	3
2-67	*K. pneumoniae*	ST105-1LV	KL81	OL13	3
2-70	*K. pneumoniae*	ST105	KL102	O1/O2v2	2
2-77	*K. pneumoniae*	ST1764-1LV	KL64	O1/O2v1	1
3-2	*K. pneumoniae*	ST15	KL24	O1/O2v1	1
3-3	*K. pneumoniae*	ST15	KL24	O1/O2v1	1
3-71	*K. pneumoniae*	ST412	KL57	O3b	2
3-74	*K. pneumoniae*	ST113	KL104	O1/O2v2	9
3-82	*K. quasipneumoniae*	ST3026	KL183	OL103	6
3-88	*K. quasipneumoniae*	ST526-1LV	KL136	O12	13
3-90	*K. pneumoniae*	ST29	KL54	O1/O2v2	2
3-92	*K. pneumoniae*	ST11	KL64	O1/O2v1	4
3-103	*K. pneumoniae*	ST45	KL24	O1/O2v1	1
4-30	*K. pneumoniae*	ST628	KL114	O3b	1
4-33	*K. pneumoniae*	ST15	KL19	O1/O2v2	1
4-55	*K. pneumoniae*	ST5133	KL118	OL13	2
4-57	*K. pneumoniae*	ST378-2LV	KL31	O3b	5
4-58	*K. pneumoniae*	ST5556	KL20	O1/O2v1	3

Among the 81 plasmids identified from the 24 *Klebsiella* strains, 28 (34.6%) were considered novel (**Supplementary Table 1**). Plasmid novelty was assessed by BLASTn comparison against the NCBI NT database, using <80% backbone sequence identity and <70% coverage as thresholds. The comparison was made with previously reported plasmids in public databases. These findings, consistent with our earlier report (Xu et al., 2024), suggest ongoing plasmid diversification and structural rearrangement within *Klebsiella* populations.

### 3.2 Antimicrobial resistance determinants in isolated *Klebsiella* strains

Fifteen of the 24 isolated *Klebsiella* strains carry antimicrobial resistance determinants on their plasmids (**Supplementary Table 1**), consistent with our previous finding that *K. pneumoniae* acquire antibiotic resistant plasmids for its antibiotic resistance (Li et al., 2018). A total of 22 such antibiotic resistant plasmids were identified. Five *Klebsiella* strains carried more than one antibiotic resistant plasmid, agreeing with the hypothesis that acquisition of multiple antibiotic resistant plasmids can lead to multidrug resistance in *Klebsiella*. To further evaluate their phenotypic resistance, minimum inhibitory concentrations (MICs) of representative antibiotics were determined for all strains. The MIC data, summarized in **Table 2**, reveal substantial variation in resistance levels among the isolates, with several strains exhibiting high-level resistance to multiple antibiotics.

**Table 2:** The antibiotic susceptibility of *Klebsiella* strains.

Strain	Antibiotics MIC value (μg/ml)												
	IPM	TGC	PME	AMP	CTX	KAN	STR	TMP	TET	CIP	GAT	MPM	SUL
1-74	S (1)	R (8)	R (8)	R(>512)	R(>512)	R(>512)	R(512)	R (32)	R(>512)	R (128)	R(128)	S (1)	S (256)
1-76	S (1)	R (16)	S (2)	R (64)	S (1)	R(>512)	S (2)	R (32)	R (32)	R (128)	R(64)	S (1)	S (256)
2-28	R(128)	R (4)	R (16)	R(>512)	R (512)	R (64)	S (4)	R (32)	S (4)	I (2)	S (1)	R (32)	S (256)
2-55	S (1)	R (4)	S (2)	R (64)	S (1)	R (256)	I (32)	R (32)	I (8)	S (1)	S (1)	S (1)	S (256)
2-59	S (1)	R (8)	S (2)	R(>512)	S (1)	R (512)	R (256)	R (32)	R(>512)	R (64)	R(64)	R (4)	S (256)
2-61	S (1)	R (8)	S (1)	R(>512)	R (512)	R(>512)	R (512)	R (32)	R (256)	R (16)	R(32)	S (1)	S (256)
2-65	R (4)	R (8)	S (2)	R(>512)	R (4)	R(>512)	S (2)	R (16)	R (256)	R (128)	R(64)	S (1)	S (256)
2-67	R(4)	R (16)	S (1)	R(>512)	I (2)	R(>512)	R (64)	R (32)	R (128)	R (128)	R(128)	S (1)	S (256)
2-70	S (1)	R (8)	S (2)	R(>512)	S (1)	R(>512)	I (32)	R (32)	R (128)	R (64)	R (64)	S (1)	S (256)
2-77	S (1)	R (8)	S (1)	R(>512)	S (1)	R (128)	S (16)	R (32)	I (8)	R (128)	R (64)	S (1)	S (256)
3-2	S (1)	R (8)	R (8)	R(>512)	R (512)	R(>512)	S (8)	R (16)	R (32)	R (256)	R (64)	S (1)	S (256)
3-3	S (1)	R (16)	R (8)	R(>512)	R (512)	R(>512)	S (8)	R (32)	R (16)	R (256)	R (32)	S (1)	S (256)
3-71	S (1)	S (1)	R (8)	R (32)	S (1)	S (2)	S (4)	R (32)	S (2)	S (1)	S (1)	S (1)	S (256)
3-74	S (1)	S (2)	S (1)	R (32)	S (1)	S (2)	S (4)	R (16)	S (1)	S (1)	S (1)	S (1)	S (256)
3-82	S (1)	S (2)	R (16)	R (64)	S (1)	S (1)	S (16)	R (32)	S (2)	S (1)	S (1)	S (1)	S (256)
3-88	R (8)	S (2)	S (1)	R (512)	R (32)	S (4)	S (4)	R (16)	R(>512)	S (1)	S (1)	R (4)	S (256)
3-90	R (4)	S (2)	S (2)	R(>512)	R (64)	R (512)	I (32)	R (32)	I (8)	R (128)	R(128)	S (1)	S (256)
3-92	R(128)	R (4)	S (1)	R(>512)	R (>512)	R(>512)	S (8)	R (16)	R(>512)	R (256)	R(256)	R(256)	S (256)
3-103	R (8)	R (4)	R (8)	R (>512)	R (64)	S (4)	S (8)	R (32)	I (8)	R (64)	R (64)	S (1)	S (256)
4-30	R (4)	R (4)	S (1)	R (>512)	R (4)	R(>512)	S (4)	R (32)	R (512)	S (1)	R (16)	R (16)	S (256)
4-33	I (2)	S (1)	S (2)	R (>512)	R (>512)	R(>512)	R (64)	R (32)	R (128)	R (8)	I (4)	S (1)	S (256)
4-55	R (16)	R (16)	R (4)	R (>512)	R (128)	R (512)	S (4)	R (32)	R (512)	S (2)	S (1)	S (1)	S (256)
4-57	I (2)	R (16)	S (1)	R (>512)	R (128)	R(>512)	R (512)	R (32)	R (256)	R (64)	R(128)	S (1)	S (256)
4-58	R (16)	R (8)	S (1)	R (>512)	R (512)	R(>512)	R (256)	R (32)	R (512)	R (128)	R(128)	R (64)	S (256)

IPM, Imipenem; TGC, Tigecycline; PME, Polymyxin E; AMP, Ampicillin; CTX, Cefotaxime; KAN, Kanamycin; STR, Streptomycin; TMP, Trimethoprim; TET, Tetracycline; CIP, Ciprofloxacin; GAT, Gatifloxacin; MPM, Meropenem; SUL, Sulfisoxazole. S, susceptible; R, resistant; I, intermediate.

**Figure 1:**
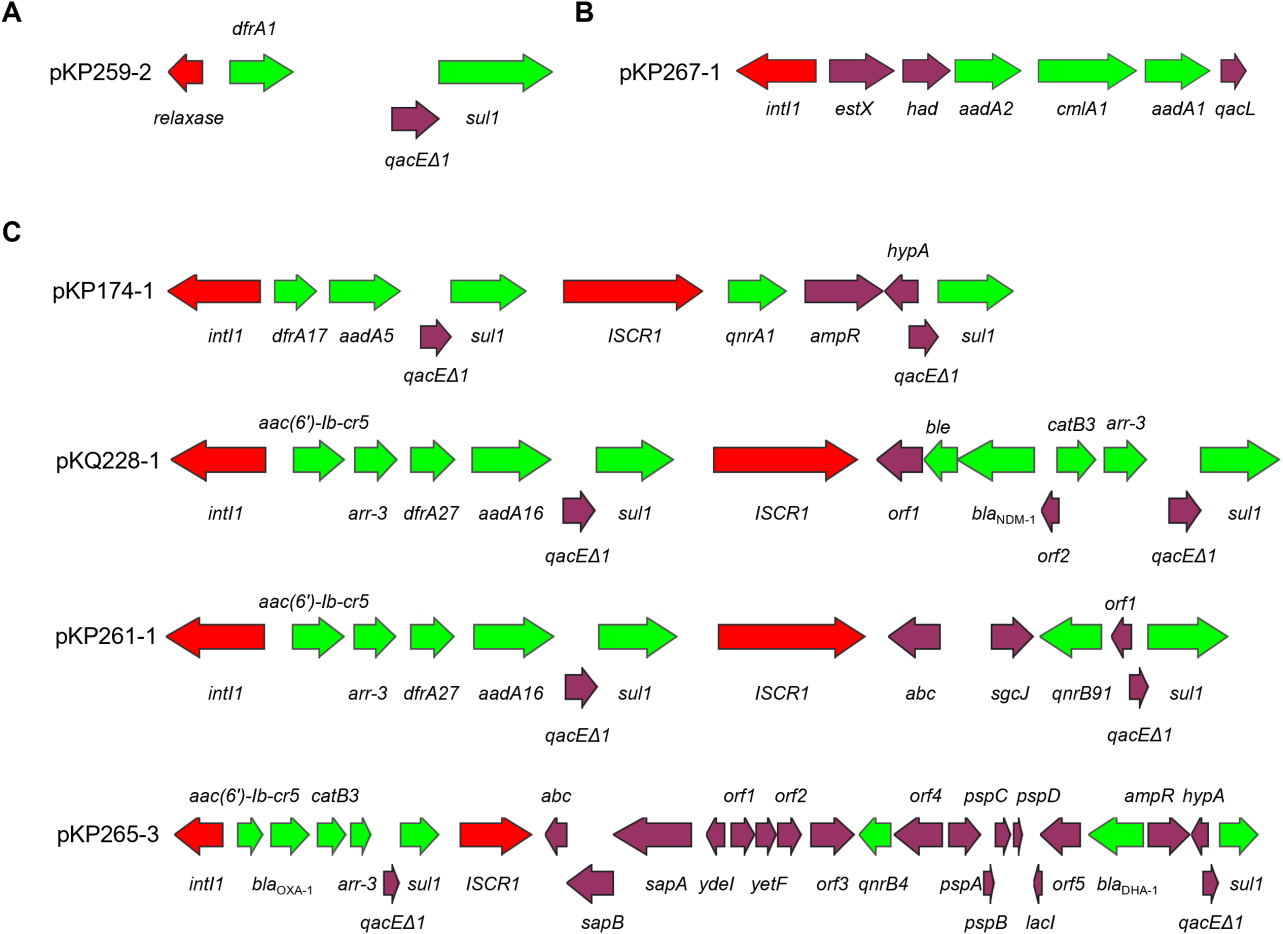
Intergrons in antibiotic resistant plasmids. Panel **(A)** pKP259–2 in which an ‘integron’ carries a relaxase-coding gene rather than *intI1*; Panel **(B)** an unconventional Class I integron in pKP267-1; Panel **(C)** complex Class I integrons found in this work. Red color indicates integrase/transposase-coding gene, green color indicates antibiotic resistance gene.

A high level prevalence of integrons were found to be associated to ARGs. Of the 22 antibiotic resistant plasmids, 12 carry integrons, four of which are complex Class I integrons (**Table 3**). Two of the integrons are unconventional. The integron carried by pKP265–1 has the exact structure of a common Class I integron, but does not carry an integrase-coding gene. Instead, it carries a relaxase-coding gene (**Figure 1A**). Whether this gene can encode an enzyme that functions similarly to an integrase is unknown. *K. pneumoniae* 2–67 carries an IncFII(K)-type antibiotic resistant plasmid p267–1 that also carries an unusual but not unprecedented Class I integron with the gene cassette array ending with *qacL* and lacking *sul1* (**Figure 1B**) (Alves et al., 2025).

**Table 3:** Antibiotic resistant plasmids.

Strain	Plasmid	Conjugative potential	New plasmid	Integron type	Gene cassette array
1-74	pKP174-1	No	No	Complex Class I integron	
	pKP174-2	Yes	No	NA	
	pKP174-3	No	Yes	NA	
2-28	pKQ228-1	Yes	Yes	Complex Class I integron	
2-55	pKP255-1	No	Yes	Class I integron	*aadA2*
2-59	pKP259-1	No	No	NA	
	pKP259-2	No	Yes	Class I integron	*dfrA27-aadA2*
2-61	pKP261-1	No	No	Complex Class I integron	
2-65	pKP265-1	Yes	No	Class I integron	*dfrA1*
	pKP265-3	No	No	Complex Class I integron	
2-67	pKP267-1	Yes	No	Class I integron	*estX-had-aadA2-cmlA1-aadA1*
3-2	pKP32-1	Yes	No	Class I integron	*dfrA12-orf1-aadA2*
3-3	pKP33-1	Yes	No	Class I integron	*dfrA12-orf1-aadA2*
3-82	pKQ382-1	Yes	Yes	NA	
3-88	pKQ388-3	Yes	Yes	NA	
	pKQ388-4	No	Yes	NA	
	pKQ388-7	No	No	NA	
3-92	pKP392-2	No	No	NA	
4-33	pKP433-1	Yes	No	Class I integron	*dfrA12-orf1-aadA2*
4-55	pKP455-1	No	Yes	NA	
4-58	pKP458-1	Yes	Yes	Class I integron	*aac(6’’)-Ib-cr5-arr-3-dfrA27-aadA16*
	pKP458-3	No	No	NA	

Four antibiotic resistant plasmids carry complex Class I integrons that are diverse in their structures (**Figure 1C**). Of particular interest is pKQ228-1, an unreported potentially conjugative plasmid that carries a very large complex Class I integron with a novel gene cassette array harboring 12 ARGs, including *bla*_NDM-1_, which is located within the IS*CR1*-associated integron region. Comparative analysis with regional *bla*_NDM-1_ plasmids carrying complex class 1 integrons revealed distinct integron structures in pKQ228-1 (**Supplementary Figure S1**). Complex Class I integrons carrying *bla*_NDM-1_ were previously reported in *Proteus mirabilis* (Li et al., 2023), *Enterobacter hormaechei* (Doualla-Bell et al., 2021), *Enterobacter cloacae* (Zhu et al., 2020), *Pseudomonas aeruginosa* (Kostyanev et al., 2020), and *Raoultella ornithinolytica* (Yu et al., 2020). To our knowledge, *bla*_NDM-1_ has not previously been reported within complex Class I integrons in *Klebsiella* species. The identification of such a structure in clinical *Klebsiella* strain may suggest a new approach of dissemination of carbapenem resistance in *Klebsiella*.

*K. pneumoniae* 1–74 hosts a conjugative pKP174–2 plasmid that carries *mcr-8.1* and *tporJ1-tmexCD1*. This plasmid may serve as a vehicle for the dissemination of resistance to polymyxin and tigecycline, both considered last-line antibiotics. Indeed, *K. pneumoniae* 1–74 is resistant to both polymyxin (MIC = 8 μg/ml) and tigecycline (MIC = 8 μg/ml). To verify transferability, a conjugation assay using *K. pneumoniae* 1-74 (donor) and *E. coli* BW25113+pRedCas9 (recipient) was performed. Transconjugants selected on MacConkey agar with streptomycin (200 µg/mL) and polymyxin E (2 µg/mL) were PCR-positive for *mcr-8.1*, confirming plasmid transfer. The transconjugant exhibited elevated MICs (Polymyxin E: 64 μg/mL; Tigecycline: 8 μg/mL), indicating that pKP174–2 confers transferable resistance. This plasmid is closely related to pHNAH8I-1 from which tigecycline-resistant *tporJ1-tmexCD1* was first identified (Lv et al., 2020). The two plasmids share conserved regions carrying *mcr-8.1*, *tmexCD1-toprJ1*, and conjugation-associated genes, highlighting their structural similarity and potential for horizontal dissemination (**Supplementary Figure S2**). However, *K. pneumoniae* strain AH8I that carried pHNAH8I-1, along with the other four reported similar strains were from chicken fecal samples (Lv et al., 2020). The identification of *K. pneumoniae* 1–74 that is from hospital samples suggested that this plasmid has now entered clinical settings and poses a direct threat to patients.

The extent of transferability of ARGs found in *Klebsiella* strains in this work, either in the form of plasmids or integrons, showed that *Klebsiella* strains quickly exchange and acquire antibiotic resistance with mobile genetic elements. This is important for the clinical setting, particularly in hospitals, because the chances of antibiotic resistant strains meet and exchange antibiotic resistance are much higher than in communities. Antibiotic resistant genetic elements can accumulate to a high level in these settings, as can be confirmed by the finding of plasmid- and integron-rich multidrug resistant *Klebsiella* strains in this work.

### 3.3 Enriched transferrable heavy metal resistance genes in hospital-origined *Klebsiella* strains

One alarming finding is the extent of heavy metal resistance genes in isolated *Klebsiella* strains. Eighteen out of 24 strains carry heavy metal resistance genes, more than that carry antibiotic resistance genes (**Table 4**). Resistance genes for eight metals and metalloids were found: chromate, tellurium, silver, copper, nickel, arsenate, cobalt, and mercury. These resistance genes were identified on 24 distinct plasmids, indicating their potential for horizontal transfer and contribution to metal resistance dissemination. This number is also larger than the 22 antibiotic resistant plasmids found. This reflects that heavy metal resistance is even more prevalent than antibiotic resistance in clinical *Klebsiella* strains isolated in this work.

**Table 4:** Metal resistant plasmids.

Strain	Plasmid	Conjugative potential	New plasmid	Antibiotic resistant plasmid	Metal resistance
1-74	pKP174-1	No	No	Yes	Cr
2-28	pKQ228-1	Yes	Yes	Yes	Te, Hg, Cr
	pKQ228-2	No	Yes	No	Ni, Co, Cu, Ag
2-55	pKP255-1	No	Yes	Yes	Te, Ag, Cu
2-59	pKP259-1	No	No	Yes	Hg, As
2-65	pKP265-1	Yes	No	Yes	Ag, Cu, As
	pKP265-3	No	No	Yes	Cr
2-70	pKP270-1	No	No	No	As, Ag, Cu
2-77	pKP277-1	No	No	No	Ag, Cu, Te
3-2	pKP32-1	Yes	No	Yes	As, Cu, Ag, Cr
3-3	pKP33-1	Yes	No	Yes	As, Cu, Ag, Cr
3-71	pKP371-1	No	No	No	Cu, Ag, Te
3-74	pKP374-1	Yes	Yes	No	As
	pKP374-2	Yes	Yes	No	As
3-82	pKQ382-1	Yes	Yes	Yes	As
	pKQ382-2	Yes	Yes	No	Co, Hg
3-88	pKQ388-1	Yes	Yes	No	Te
	pKQ388-7	No	No	Yes	Co
3-90	pKP390-1	Yes	No	No	Te
3-92	pKP392-1	Yes	No	No	Hg
	pKP392-3	Yes	Yes	No	Co
4-30	pKP430-1	Yes	Yes	No	As, Cu, Ag
4-33	pKP433-1	Yes	No	Yes	As, Cu, Ag, Cr
4-55	pKP455-1	No	Yes	Yes	As, Cu, Ag

The enriched transferrable heavy metal resistance genes in *Klebsiella* showed that metal resistance is even worse than, or at least comparable with, antibiotic resistance in the hospital setting studied in this work. This makes sense, because in hospitals heavy metals are commonly used for medication. In the case of traditional Chinese medicine, herbs are also often contaminated by heavy metals (Yang et al., 2018). This may explain the level of heavy metal resistance observed in this study. Previous research has shown that hospital effluents often contain heavy metals and other pollutants. For example, analyses of hospital wastewater from hospitals in Indonesia during the COVID-19 pandemic, detected heavy metals and pharmaceuticals that may exert co-selection pressure on bacteria (Sakina et al., 2023). Although specific data for Chinese hospitals remain limited, these findings suggest that hospital-originated *Klebsiella* strains may be exposed to both antibiotic and heavy metal selective pressures, warranting further investigation.

The level of transferability for metal resistance genes is high. Not only are they hosted by plasmids, 58.3% (14) of these plasmids also carry transconjugation gene cassettes (**Table 4**). This prompted us to wonder whether heavy metal resistance, similarly to antibiotic resistance, is also enriched in clinical *Klebsiella* strains under constant selection pressure, and induced by high transferability of the heavy metal resistance determinants.

**Figure 2:**
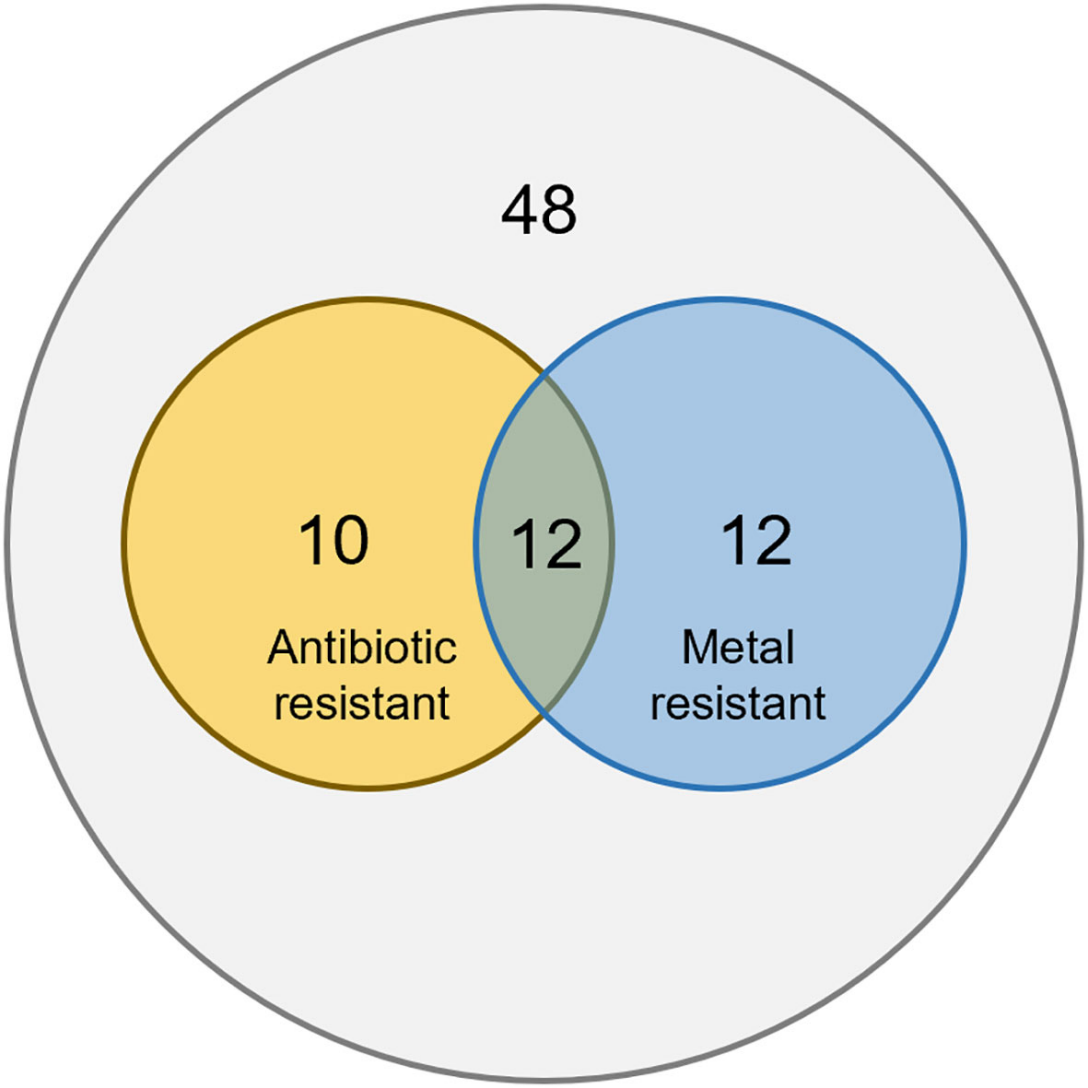
Venn map on plasmids in antibiotic and metal resistance.

Twelve plasmids were found to host both antibiotic resistance genes and heavy metal resistance genes. This accounts for 54.5% of antibiotic resistant plasmids, and 50% of heavy metal resistant plasmids. This suggests co-selection of antibiotic and metal resistance on plasmids (**Figure 2**). Previous studies have shown that exposure to antibiotics or heavy metals can promote co-selection of resistance determinants across different stressors (Wales and Davies, 2015; Mustafa et al., 2021), indicating that environmental contaminants can drive the aggregation and dissemination of multiple resistance traits. We suspect that antibiotic or heavy metal stress can induce the evolution and aggregation of not only the resistance against the exposed stress, but also resistance against the other stress.

**Figure 3:**
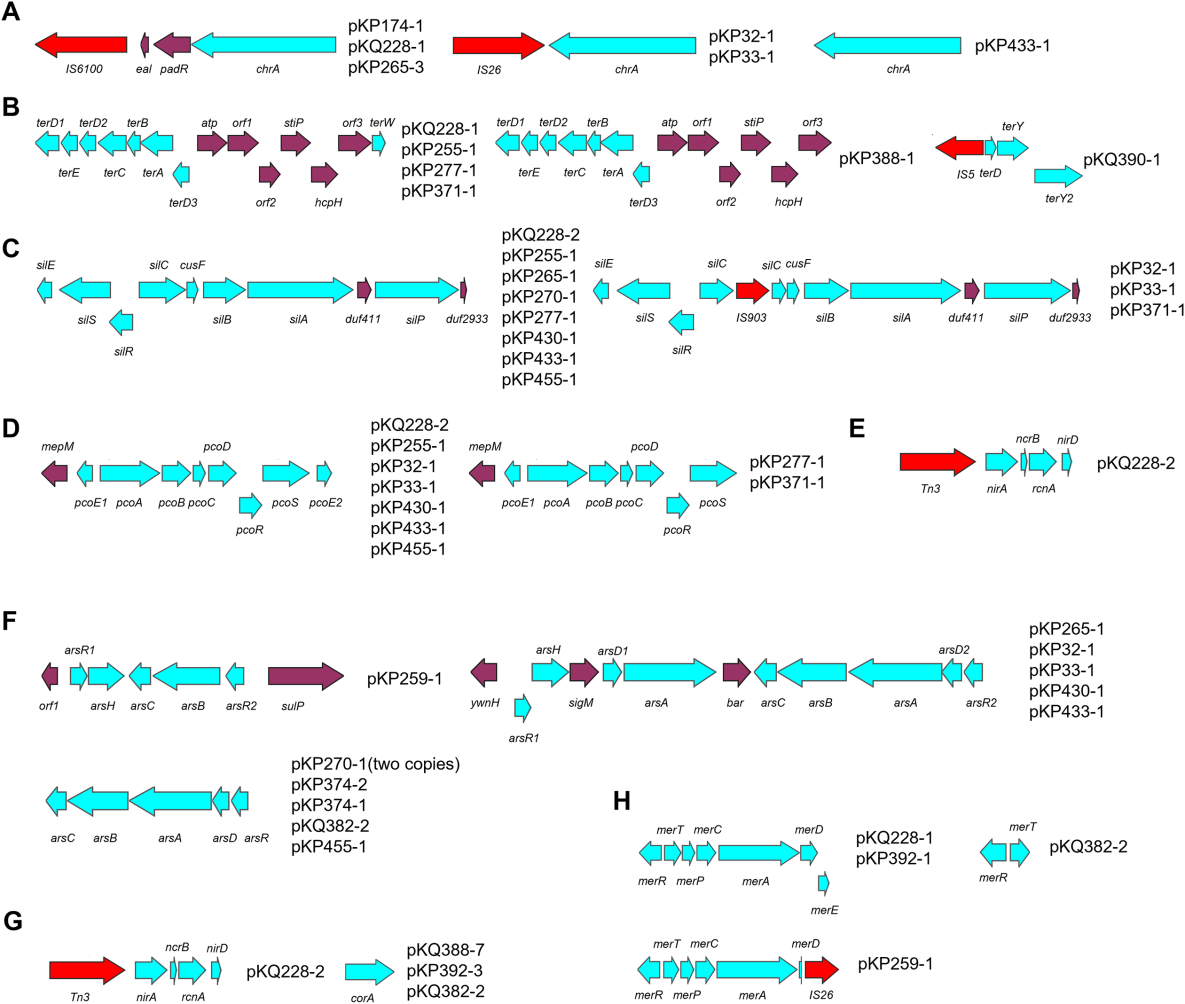
Plasmid-borne heavy metal resistance gene clusters. Panel **(A)** Chromate-resistant gene clusters; Panel **(B)** Tellurium-resistant gene clusters; Panel **(C)** Silver-resistant gene clusters; Panel **(D)** Copper-resistant gene clusters; Panel **(E)** Nickel-resistant gene cluster; Panel **(F)** Arsenate-resistant gene clusters; Panel **(G)** Cobalt-resistant gene clusters; Panel **(H)** Mercury-resistant gene clusters. Red coloor indicates integrase/transposase-coding gene, blue color indicates heavy metal resistance gene, purple color indicates others genes. Strain 3–82 harbors plasmid pKQ382-2, which contains an incomplete mercury resistance cluster.

Heavy metal resistance genes were organized in a limited number of gene clusters in the metal resistant plasmids found (**Figure 3**). This is another piece of evidence suggesting the transferability of heavy metal resistance among *Klesiella* strains. Identical metal resistant clusters could be found in different plasmid types, under different genetic contexts, in different strains, and even in different species (*K. pneumoniae* and *K. quasipneumoniae*).

The genetic organization of heavy metal resistance gene clusters were inspected. All chromate resistance was generally associated with the presence of *chrA* that encodes a membrane-bound efflux pump (Caballero-Flores et al., 2012). Three genetic structures were found associated with *chrA*, with a *IS6100-eal-padR-chrA* structure present in both *K. pneumoniae* and *K. quasipneumoniae* (**Figure 3A**). Three conserved genetic structures were found to be related to tellurium resistance, with the gene cluster in pKP388–1 being a variant of that in pKP228-1 (**Figure 3B**). All silver-resistant gene clusters are similar. Those present in pKP32-1, pKP33-1, and pKP371–1 are *IS903* insertion variants of those found in pKQ228-2. In hospitals, silver is widely used as disinfectants for its antibacterial properties (Li and Xin, 2025). Therefore, the prevalence of its resistance in hospital wastewaters is not a surprise. Similarly, a copper resistant gene cluster (like in pKP228-2) and its *ΔpcoE2* variant led to copper resistance. Both nickel and cobalt resistance were encoded in the same gene cluster in pKQ228-2 (**Figure 3E** and 3G), whereas in three plasmids nonspecific transporter *corA* may be responsible for cobalt resistance (Gibson et al., 1991). Arsenic resistance was prevalent and three forms of gene clusters were found to encode arsenic resistance (**Figure 3F**). Arsenic trioxide is a well-known medicine for acute promyelocytic leukemia, explaining the prevalence of its resistance mechanisms (Paul et al., 2023). Resistance gene clusters for mercury, an environmental pollution and widely used dental filling (Iqbal and Asmat, 2012), was also found in four plasmids in three formats. While some resistant strains lacked the corresponding genes, the observed associations are supported by previous reports and suggest potential genotype–phenotype correlations.

Phenotypic analysis of heavy metal resistance was performed for *Klebsiella* strains that host heavy metal resistance genes, except for arsenic resistance because arsenic-containing compounds are heavily regulated and cannot be purchased. A high level of concordance was found on the carriage of heavy metal resistance and heavy metal resistance phenotypes (**Supplementary Figure S3**). The detailed MIC results for different heavy metals are summarized in **Table 5**, further supporting the observed genotype–phenotype consistency. Several exceptions exist: strains 2-28, 2-65, 3-2, and 4–33 carry chromate resistance genes but did not show chromate resistance; strain 3–82 carries mercury resistance genes but didn’t show mercury resistance. The case for 3-82 (pKQ382-2) should be due to the incompleteness of the mercury resistance cluster in strain 3-82 (**Figure 3**).

**Table 5:** The heavy metals susceptibility of *Klebsiella* strains.

Strain	Heavy metals MIC value (μg/ml)						
	Chromate	Silver	Cobalt	Nickel	Tellurium	Copper	Mercury
1-74	128			>512			
2-28	128	16	256		8	>512	16
2-55		16			64	>512	
2-59							16
2-65		16				>512	
2-70		16				>512	
2-77		16			128	>512	
3-2	128	16				>512	
3-3	128	16				>512	
3-71		16			64	>512	
3-82			256				
3-88			256		32		
3-90					8		
3-92			256				32
4-30		16				>512	
4-33		16				>512	
4-55		16				>512	

The analysis of antibiotic resistance and heavy metal resistance can provide answers to the questions we aimed to answer in this work. Plasmids play a major role in heavy metal resistance in *Klebsiella* species, as heavy metal resistance plasmids are similarly prevalent in the clinical *Klebsiella* strains studied. Antibiotic resistance and heavy metal resistance showed a high level of correlation, and co-selection of the two resistance types could be taking place. Similarly to antibiotic resistance plasmids, a large portion (45.8%) of heavy metal resistance plasmids are new. This work showed how little we know about the structure and types of antibiotic and heavy metal resistance plasmids in *Klebsiella*, and provides rationale for truly large scale surveillance. We believe this work serves as a starting point for such investigations.

## Data Availability

The datasets presented in this study can be found in online repositories. The names of the repository/repositories and accession number(s) can be found below: https://www.ncbi.nlm.nih.gov/genbank/, PRJNA1273660.

## Supplementary material

Supplementary Figure 1 Comparative analysis of the *bla*_NDM-1_ regions of plasmid pKQ228–1 and representative *bla*_NDM-1_-harboring plasmids carring complex class 1 integrons. Arrows represent open reading frames, colored according to functional annotation.

Supplementary Figure 2 Comparative plasmid map of pKP174–2 and pHNAH8I-1. Resistance genes (*mcr-8.1, tmexCD1-toprJ1*) and conjugation-related modules are highlighted with distinct colors and symbols.

Supplementary Figure 3 Heavy metal resistance phenotypes. Panel A. Chromate resistance; Panel B. Silver resistance; Panel C. Cobalt resistance; Panel D. Nickel resistance; Panel E. Tellurium resistance; Panel F. Copper resistance; Panel G. Mercury resistance. *K. pneumoniae* ATCC13883 was used as the control strain. All *K. pneumoniae* ATCC13883 figures at zero heavy metal concentrations are the same plate, as they are essentially the same experiment (no metal, the same strain).
