# Supplementary figures and images for: Prevalent and diverse new plasmid-encoded heavy metal and antimicrobial resistance in Klebsiella strains isolated from hospital wastewater
Source: Front Cell Infect Microbiol. 2025 Nov 25;15:1653886. doi: 10.3389/fcimb.2025.1653886 (PMC12685809; doi:10.3389/fcimb.2025.1653886)

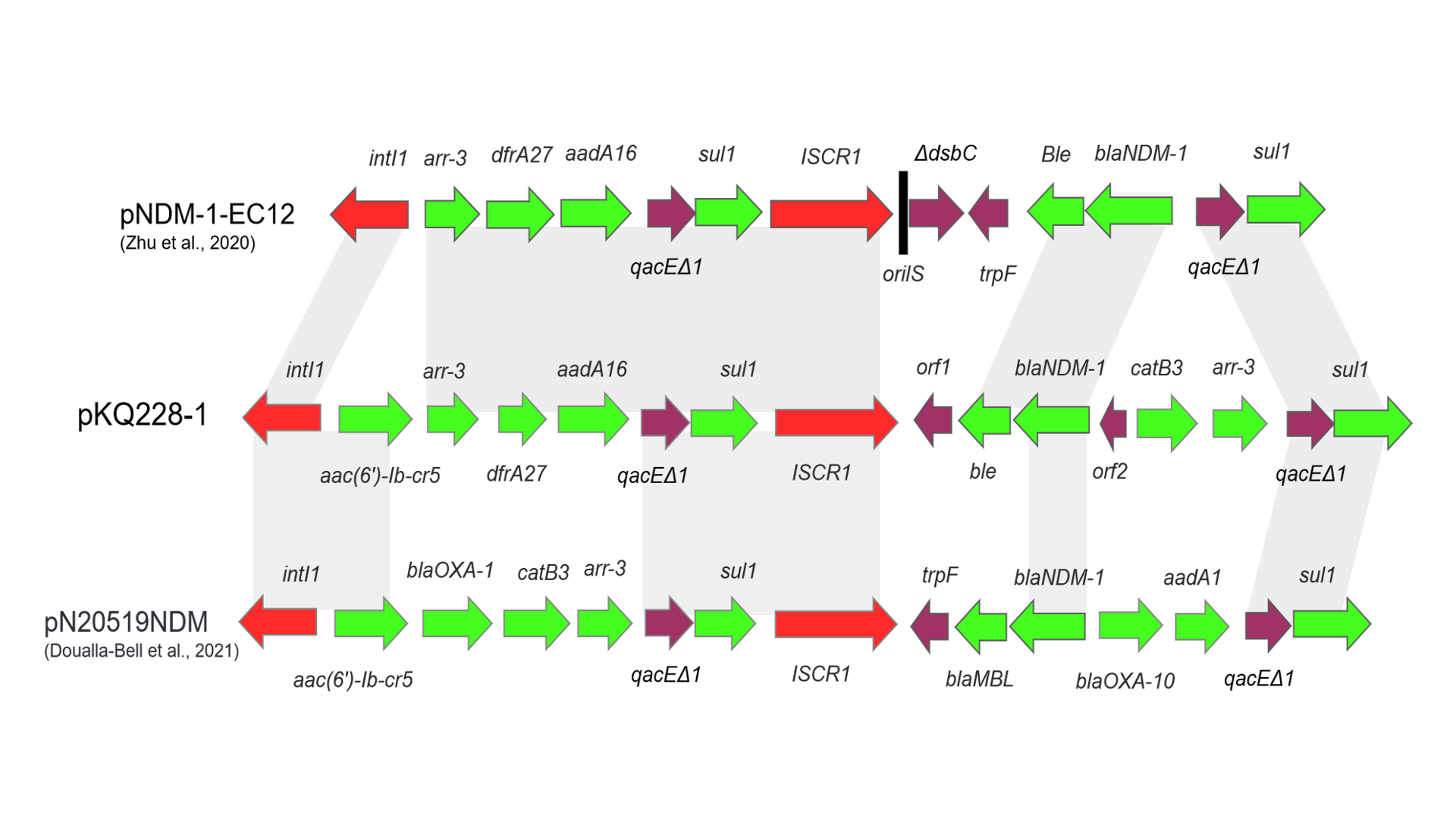

Supplement: Supplementary Figure 1 — Comparative analysis of the blaNDM-1 regions of plasmid pKQ228–1 and representative blaNDM-1-harboring plasmids carring complex class 1 integrons. Arrows represent open reading frames, colored according to functional annotation. [file Image1.tif]

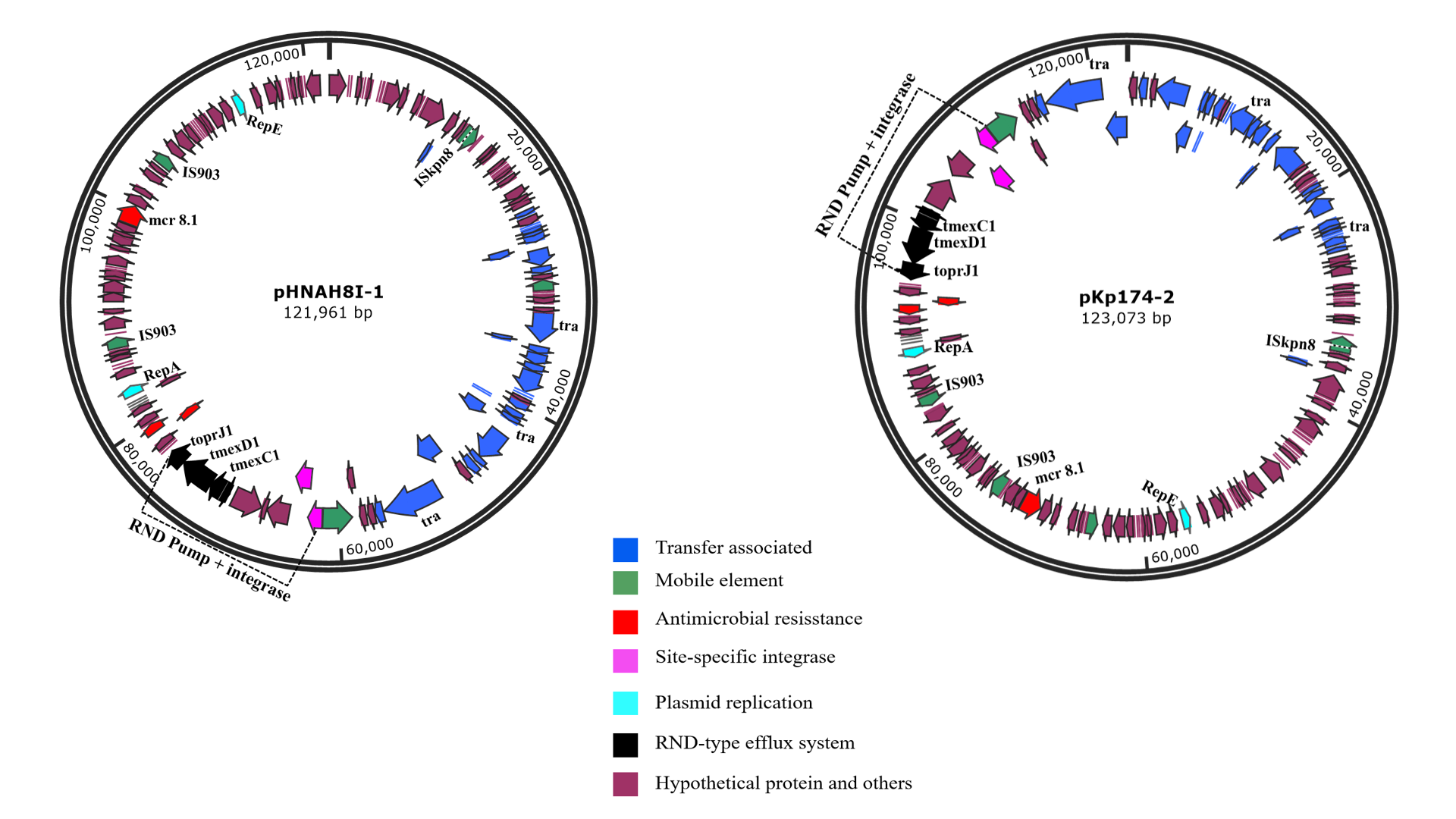

Supplement: Supplementary Figure 2 — Comparative plasmid map of pKP174–2 and pHNAH8I-1. Resistance genes (mcr-8.1, tmexCD1-toprJ1) and conjugation-related modules are highlighted with distinct colors and symbols. [file Image2.tif]

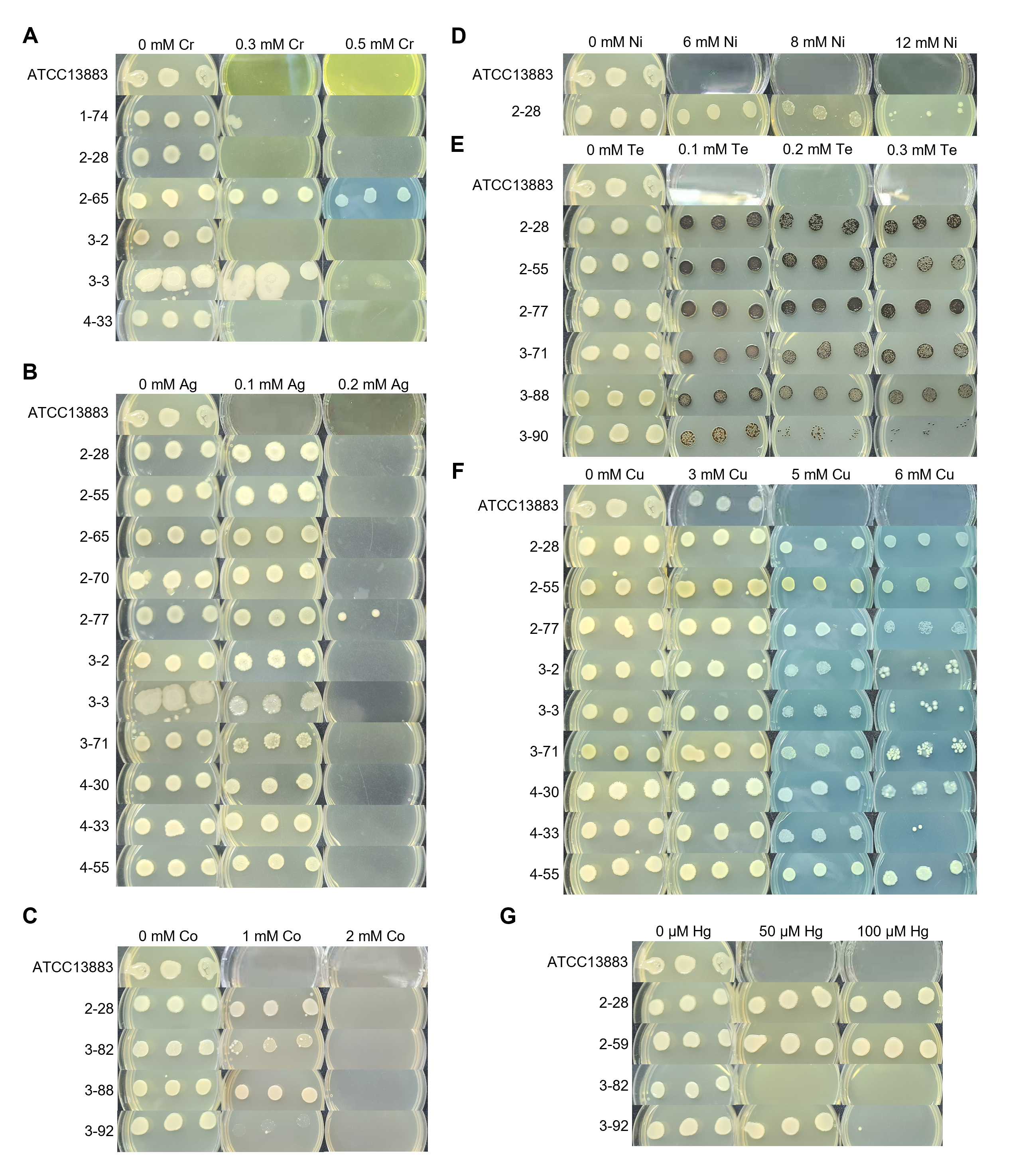

Supplement: Supplementary Figure 3 — Heavy metal resistance phenotypes. Panel A. Chromate resistance; Panel B. Silver resistance; Panel C. Cobalt resistance; Panel D. Nickel resistance; Panel E. Tellurium resistance; Panel F. Copper resistance; Panel G. Mercury resistance. K. pneumoniae ATCC13883 was used as the control strain. All K. pneumoniae ATCC13883 figures at zero heavy metal concentrations are the same plate, as they are essentially the same experiment (no metal, the same strain). [file Image3.tif]
